# The Effect of Ultrasonic Peening Treatment on Fatigue Performance of Welded Joints

**DOI:** 10.3390/ma9060471

**Published:** 2016-06-14

**Authors:** Xiaohui Zhao, Mingyi Wang, Zhiqiang Zhang, Yu Liu

**Affiliations:** 1 Key Laboratory of Automobile Materials, School of Materials Science and Engineering, Jilin University, Changchun 130025, China; zhaoxiaohui@jlu.edu.cn (X.Z.); wangmpaper@gmail.com (M.W.); 2 School of Mechanical Science and Engineering, Jilin University, Changchun 130025, China; liuyuu@jlu.edu.cn

**Keywords:** ultrasonic peening treatment, stress relief, welded joint, fatigue, stress concentration

## Abstract

Ultrasonic peening treatment (UPT) as a method of severe plastic deformation was used to treat cruciform welded joints of Q345 steel. The application of UPT achieves material surface nanocrystallization of the peening zone, reduces stress concentration, and produces residual compressive stresses at the welded toe. Micro-structure, hardness, stress relief, *S*-*N* curve, and the fatigue fracture mechanism of cruciform welded joint of Q345 steel, both before and after UPT, were analyzed in detail. The main results show that: stress concentration and residual tensile stress are the main reasons to reduce fatigue strength of cruciform welded joints. The fatigue life of cruciform welded joints is improved for surface hardening, compressive stress, and grain refinement by UPT. Residual compressive stress caused by UPT is released with the increase of fatigue life. A very significant fatigue strength improvement happens when UPT is replenished repeatedly after a certain number of cycles.

## 1. Introduction

Welding has been widely used in many aspects of industrial production as a main method of connecting mechanical parts. Usually, a welded joint has a higher stress concentration and a larger tensile residual stress at the weld toe, which will significantly reduce the fatigue life of the welded joint. Therefore, fatigue failure is one of the most important failure cases for welded joints [1,2,3]. It is well established that fatigue strength of welded joints can be enhanced by producing compressive residual stress, reducing stress concentration, increasing hardness, and forming nanocrystalline structures. Currently, the existing methods of improving fatigue performance consist of Tungsten inert gas (TIG) dressing, common hammering, weld toe grinding, a spraying method, shot peening, and so on [4,5,6,7,8]. TIG dressing has always been a simple and effective method. Previous studies [9] have shown that fatigue strength can increase by 37% after TIG dressing under constant amplitude loading. In contrast, the effect of TIG dressing is not very good under variable-amplitude loading, because compressive stress is easily relaxed when a high value stress occurs at a particular moment. Additionally, improper operation also can cause some side effects for TIG dressing [10]. The common hammering method has many disadvantages, such as low efficiency, large noise, great labor intensity, poor controllability, and instable effect [11], and, as such, is not suited for standard-processing. The spraying method can effectively reduce stress concentration and improve surface properties, but the bonding strength between the layer and the matrix is weaker, and a fatigue crack can easily form at the bonding position under the high stress level [12]. Weld toe grinding can effectively improve fatigue strength of a welded joint [13,14], but static strength may decrease after grinding. Shot peening is one of the most widely used surface enhancing methods to improve fatigue strength [15,16,17]. Fatigue properties of the parent metal treated by shot peening have been documented in detail by Bagherifard *et al.* and Hassani-Gangaraj *et al*. [18,19]. Fatigue life is substantially improved after shot peening treatment, but shot peening cannot effectively deal with the position of stress concentration on a cruciform welded joint due to the uncontrollability of pill granules. As such, the improvement of fatigue life for a cruciform welded joint still cannot depend entirely on shot peening. Furthermore, there are many complicated fillet welds for a large welding structure; utilizing the shot peening treatment for these welded joints is not realistic [20].

In view of the above analysis, UPT was used to treat cruciform welded joints of Q345 steel. Stress concentration at the position of the weld toe can be significantly reduced [21,22,23,24]. A thin layer of nanocrystals will be formed, and residual tensile stress can be changed into residual compressive stress on the peening surface. These effects are useful to stop or prevent crack propagation.

In this study, the relevant characteristics surrounding the ability of UPT to improve fatigue performance of cruciform welded joints of Q345 steel will be studied. The effect of residual compressive stress caused by UPT on fatigue life will be discussed in a detailed analysis. Meanwhile, the roles of nanocrystalline structures, surface hardening, and stress concentration in the process of fatigue fracture will also be further discussed.

## 2. Material and Experimental Procedures

### 2.1. Joint Type and Experimental Material

**Figure 1:**
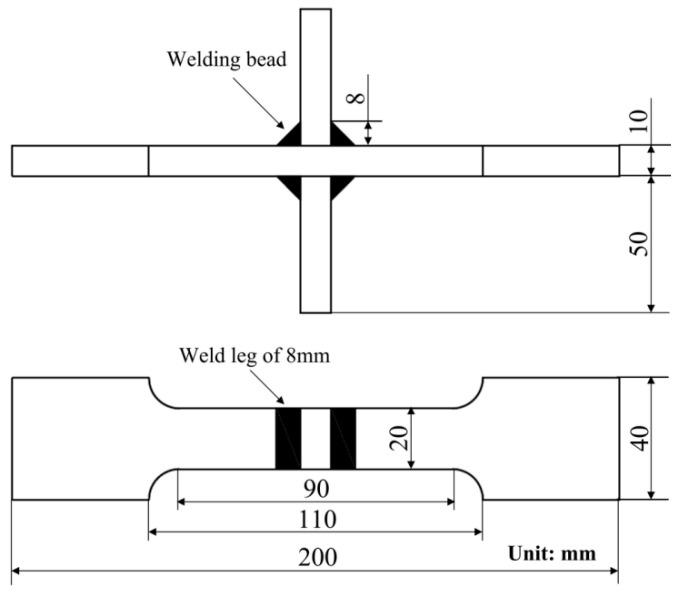
The geometrical characteristics of the fatigue specimens.

The fatigue specimens were cruciform welded joints of Q345 steel (Angang Steel Company Limited, Anshan, China), whose geometrical characteristics are shown in Figure 1. The main chemical composition (wt %) of Q345 steel is: C ≤ 0.18, Mn ≤ 1.70, Si ≤ 0.50, P ≤ 0.030, S ≤ 0.025, and Al ≥ 0.015. The mechanical properties of Q345 steel are shown in Table 1. Q345 is a kind of low alloy steel, which has good toughness and ductility. CO_2_ arc welding is the connecting method.

**Figure 2:**
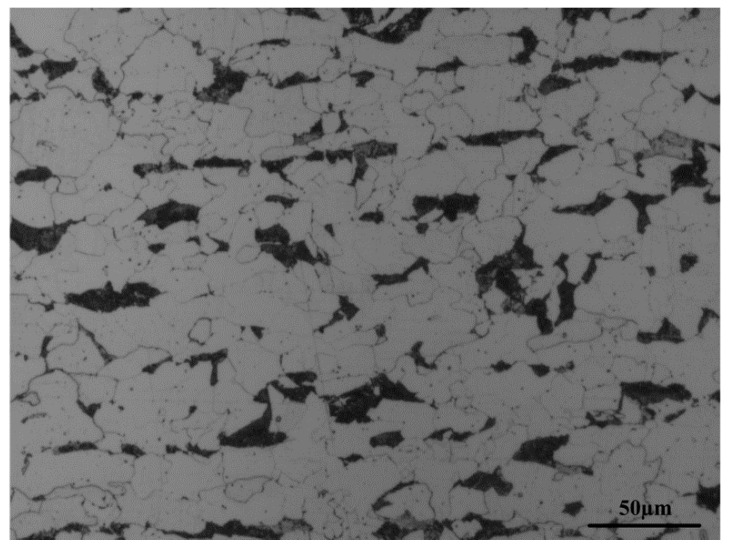
Metallographic structure of Q345 steel.

The metallographic structure of Q345 steel, which contains a large amount of white ferrite and a small amount of black pearlite, is shown in Figure 2. The metallographic structure was relatively uniform and no defects exist.

### 2.2. Surface Strengthening Treatment

HJ-III type ultrasonic peening equipment produced by Tianjin University, in China, was used to treat the weld toe of the cruciform welded joints. After UPT, the larger stress concentration of the weld toe was reduced, and the residual compressive stress formed at the weld toe. Meanwhile, the high-frequency impacts refined the grains. Thus, with the decrease ingrain size, the material hardness increased gradually. The detailed parameters of UPT are shown in Table 2. In order to ensure peening effects, multiple treatments were implemented.

### 2.3. Fatigue Testing Scheme

Fatigue tests of cruciform welded joints were performed. Under tension-tension constant amplitude loading mode (sine wave loading), each welded joint was tested at five stress levels with stress ratio *R* = 0.1 at room temperature in air environment. Fatigue tests were conducted on a 200 kN high frequency fatigue testing machine (CIMACH, Changchun, China) with static load error within a full measuring range between ±0.2% and dynamic load error between ±2%. The frequency used was the resonance frequency determined by the fatigue specimen and testing machine. The value of the frequency in these fatigue tests is about 100–120 Hz. The detailed fatigue testing scheme was as follows:

(1) Fatigue tests of two kinds of welded joints (as-welded joint and UPT-welded joint) were carried out. The effect of UPT can be observed through the *S-N* curves.
(2) UPT-welded joints were given an extra supplement of ultrasonic peening during the process of fatigue tests every 50,000, 100,000, and 150,000 cycles, respectively. The effect of residual compression stress release on fatigue life can be studied by this way.

## 3. Results and Discussion

### 3.1. Surface Strengthening Mechanism of UPT

**Figure 3:**
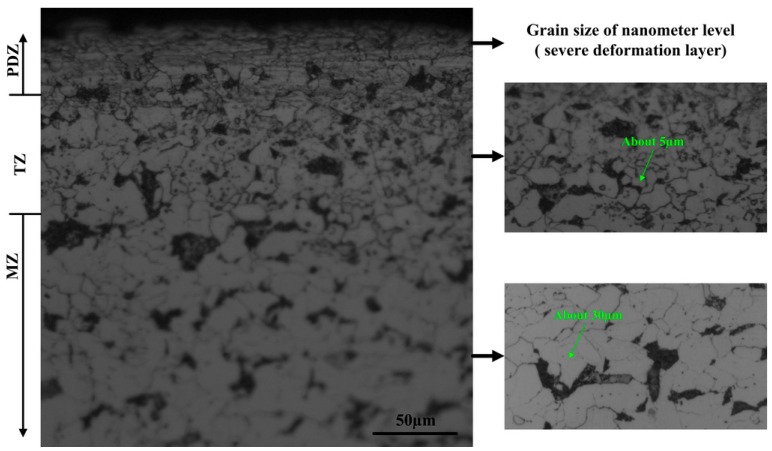
Microstructure and deformation layer after Ultrasonic peening treatment (UPT) (PDZ: Plastic deformation zone; TZ: Transition zone; MZ: Matrix zone).

The OLS3000 laser confocal microscope (ZEISS, Oberkochen, Germany) was adopted to observe microstructures and the deformation layer after UPT (see Figure 3). After UPT the specimens presented three areas: the Plastic Deformation Zone (PDZ), the Transition Zone (TZ), and the Matrix Zone (MZ). Among them, PDZ mainly contained fine grains with different orientations. The average size of fine grains had reached a nanometer level. PDZ is the area affected directly by the high speed ultrasonic peening. Microstructure characteristics of the TZ are between the PDZ and the MZ; the grain size of the TZ was bigger than that of the PDZ. With the increase indepth, the grain size of the TZ will be the same with that of the MZ.

It can be observed from Figure 3 that the thickness of the PDZ is about 40 µm. Because the plastic deformation layer is formed and the grain size is refined to the nanoscale, the strength and hardness of the material surface will be significantly improved after UPT. Meanwhile, compression stress caused by static loading pressure during the process of UPT is reserved through plastic deformation in a certain depth from the material surface. Residual compressive stress is one of the most important factors of improving fatigue performance.

### 3.2. Hardness Analysis

**Figure 4:**
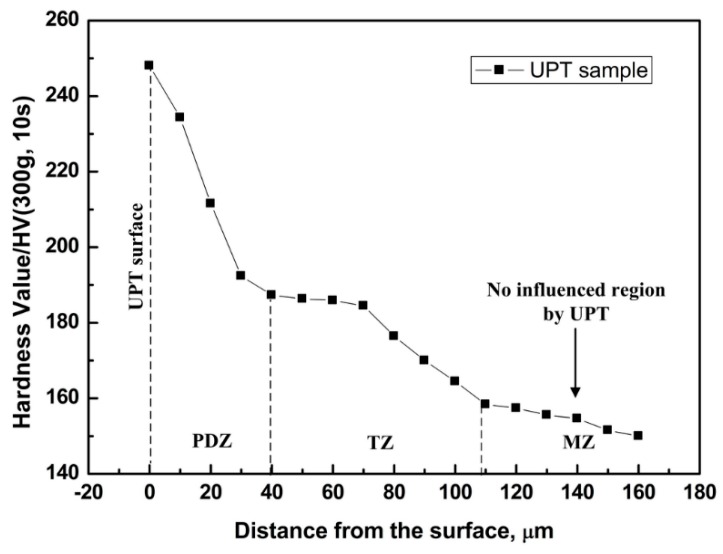
The variation trend of hardness values after UPT from surface to interior.

Models for MH-3 Vickers hardness tester (Shanghai Hengqi Precision Machinery Plant, Shanghai, China) were adopted to measure the hardness values. The hardness values after UPT were measured along the direction of thickness (from surface to interior). The loading was 300 g and lasting time was 10 s. The variation trend of hardness values are shown in Figure 4. Hardness values on the material surface after UPT were larger. Hardness value decreases as the depth increases. Eventually, the hardness values tended to reach a stable level. The increase of hardness values was caused by both grain refinement and work-hardening. Hardness value of the Q345 steel before UPT was about 150 HV. After UPT, the largest hardness value was 246 HV, which increased by 64% comparing with the value before UPT.

### 3.3. Fatigue Life Analysis

In this study, nominal stress (the load divided bythe flat part cross-section of the specimen) was adopted. Nominal stress level and fatigue life together determine the fatigue performance of a specimen. The relationship between nominal stress range (Δσ) and fatigue life (*N*) is represented in Equation (1). The curve based on the Equation (1) is the traditional *S-N* curve. Where, σ_max_ and σ_min_ is the maximum nominal stress and minimum nominal stress in a cycle, respectively.

(1) $$N=\frac{C}{\Delta \sigma^{m}}$$

(2) $$\Delta \sigma =\sigma_{\max}-\sigma_{\min}$$

**Figure 5:**
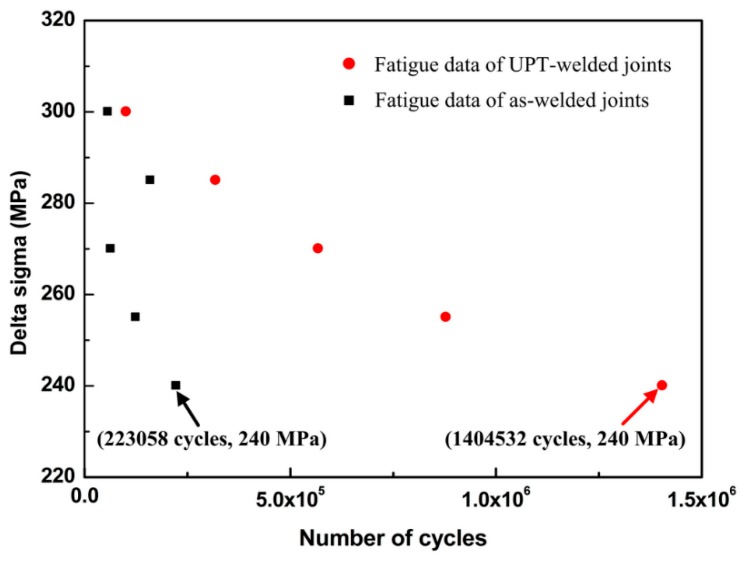
Fatigue data of UPT-welded joints and as-welded joints.

According to the Equations (1) and (2), fatigue data of UPT-welded joints and as-welded joints are presented in Figure 5.

In Figure 5, the fatigue strength of UPT-welded joints has been improved with respect to that of the not-peened samples (as-welded joints). Notably, with the decrease instress level, the effect of UPT for improving the fatigue life of welded joints is better. When the nominal stress range (delta sigma) was 240 MPa, the fatigue lives of the as-welded joint and UPT-welded joint were 223,058 cycles and 1,404,532, respectively. The fatigue life of the UPT-joint was 6.3 times that of the as-welded joint. However, the fatigue life of the UPT-welded joint was close to that of the as-welded joint under a high stress level. For example, when delta sigma was 300 MPa, the fatigue lives of the as-welded joint and UPT-welded joint were essentially the same. The reason for the above phenomenon is that residual compressive stress can be preserved under alow stress level, but residual compressive stress will be released under ahigh stress level. Therefore, the effect of UPT is better for the improvement of fatigue life under alow stress level.

### 3.4. The Effect of Stress Release on Fatigue Life

**Figure 6:**
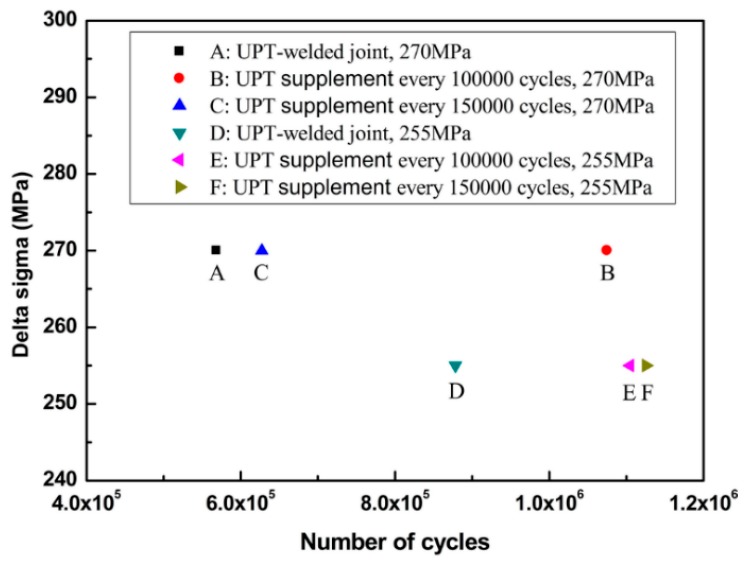
Fatigue life of UPT-welded joints and UPT supplement joints.

Residual compressive stress caused by UPT will be relaxed with an increase of fatigue life. Especially for the welded structure parts during serving, the release of residual compressive stress caused by UPT will make the effect of ultrasonic peening disappear. Thus, the welded structure parts during serving will be very dangerous. So, if UPT is continuously reapplied every few cycles, the compressive stress will have been preserved, and thus the fatigue life of the welded joints will also be further prolonged. Given this, five samples (A, B, C, D, and E) were prepared and subjected to the fatigue tests. Sample A was a UPT-welded joint under the nominal stress range of 270 MPa; Sample B was a UPT-welded joint that had ultrasonic peening reapplied every 100,000 cycles under the nominal stress range of 270 MPa; Sample C was a UPT-welded joint that had ultrasonic peening reapplied every 150,000 cycles under the nominal stress range of 270 MPa; Sample D was a UPT-welded joint under the nominal stress range of 255 MPa; Sample E was a UPT-welded joint that had ultrasonic peening reapplied every 100,000 cycles under the nominal stress range of 255 MPa; Sample F was a UPT-welded joint that had peening reapplied every 150,000 cycles under the nominal stress range of 255 MPa. Fatigue data of the six samples are shown in Table 3 and Figure 6.

From Table 3 and Figure 6 we can obviously ascertain that UPT reapplications are beneficial to the improvement of fatigue life. Under the nominal stress range of 270 MPa (high stress level), the effect of UPT reapplications every 100,000 cycles (Sample B) was better than that of UPT reapplications every 150,000 cycles (Sample C). The fatigue lives of Sample B and Sample C were 1.9 times and 1.1 times that of Sample A, respectively. The above mentioned results indicate that residual compressive stress is not completely relaxed when fatigue life reaches 100,000 cycles. At this point, the UPT reapplicationswill result inthe residual compressive stress being maintained at the position of the weld toe, thereby improving fatigue life significantly. However, residual compressive stress has been observed to be completely relaxed when the fatigue life reaches 150,000 cycles, and the tensile stress is potentially still a factor at this point, so the improvement of the fatigue life is not obvious. For the welded structure parts during serving, the timely reapplication of UPT can effectively avoid the occurrence of safety accidents.

Under the nominal stress range of 255 MPa (low stress level), the effect of UPT reapplications every 100,000 cycles (Sample E) was basically the same with that of UPT reapplications every 150,000 cycles (Sample F). The fatigue lives of Sample E and Sample F were 1.26 times and 1.28 times that of Sample D, respectively. These results indicate that the release rate of the residual compressive stress is slower under the low stress level. UPT reapplications under the low stress level were not as important as they were under the high stress level.

### 3.5. Fatigue Fracture of Welded Joint before and after UPT

**Figure 7:**
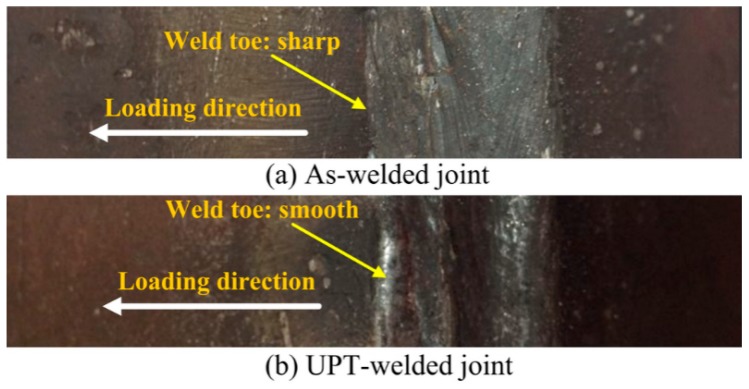
Stress concentration of (**a**) an as-welded joint; and (**b**) UPT-welded joint.

In this study, fatigue failure means that a crack can be observed during fatigue tests. For as-welded joints, stress concentration and tensile stress exist at the weld toe, as such, the fatigue crack occurs at the weld toe. After UPT, the sharp weld toe is changed into a smooth weld toe, so stress concentration at the position of the weld toe can be significantly reduced (see Figure 7). The fatigue strength of UPT-welded joints is further improved for the reduction of stress concentration.

**Figure 8:**
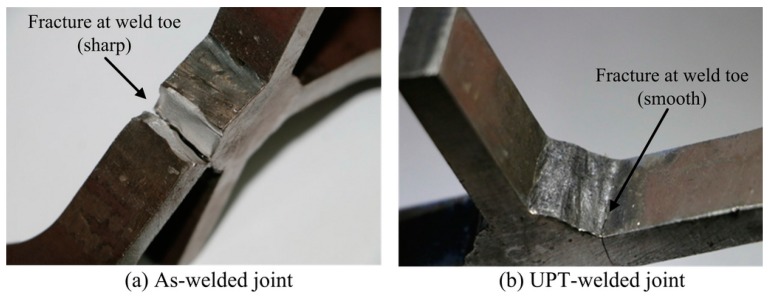
The fracture positions of (**a**) an as-welded joint; and (**b**) UPT-welded joint.

Figure 8 shows the fracture positions of an as-welded joint and UPT-welded joint. Comparing Figure 8a with Figure 8b, the positions of the fatigue fractures are at the weld toe for both the as-welded joint and UPT-welded joint. However, the fatigue crack initiation of the UPT-welded joint requires a long time for the smooth shape of the weld toe, and the fatigue life of the UPT-welded joint also is improved. For the as-welded joint, the process of the fatigue crack initiation almost does not exist for the sharp shape of the weld toe, and fatigue life of the as-welded joint is directly decided by the process of the crack propagation, so the fatigue life of the as-welded joint is lower. In addition, the as-welded joint has fractured completely for the fast crack propagation related testing conditions (see Figure 8a). However, the UPT-welded joint only produced a short crack under the condition of residual compressive stress and smooth weld toe.

**Figure 9:**
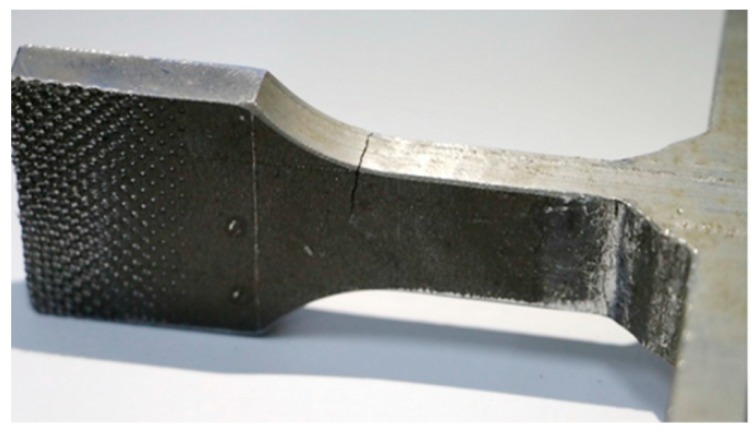
Fracture position of a welded joint of a sample that received UPT reapplications (Number of cycles: 1,104,878 cycles, delta sigma: 255 MPa).

For the welded joints with UPT reapplications, the weld toe always maintained during the residual compressive stress, therefore fatigue fracturing has not occurred at the weld toe, and instead another weak point of the sample has now become the position of crack initiation (see Figure 9). Although fracturing may happen in other weak points, the fatigue life of the welded joints after UPT reapplicationsis still obviously improved. Figure 9 shows that the position of fatigue crack initiation has changed from the weld toe to the circular arc transition position of a sample after UPT reapplications.

## 4. Conclusions

Ultrasonic peening treatment was used to improve the fatigue performance of cruciform welded joints of Q345 steel. By analyzing micro-structure, hardness, stress relief, *S-N* curve, and fatigue fracture, we can draw the following conclusions:

(1) Ultrasonic peening treatment can achieve nanocrystallization on the surface of the peening sample, reduce stress concentration, and form residual compressive stresses at the weld toe.
(2) Stress concentration and residual tensile stress are the main reasons to reduce fatigue strength of cruciform welded joints.
(3) Residual compressive stress caused by ultrasonic peening treatment will be released with the increase of fatigue life. A very significant fatigue strength improvement occurs when the ultrasonic peening treatment is reapplied repeatedly after a certain number of cycles.

## Figures and Tables

**Table 1:** The mechanical properties of Q345 steel.

Material	Yield Strength/MPa	Ultimate Tensile Strength/MPa	Elongation Rate/%	HV
Q345 steel	≥345	490–675	≥22	150

**Table 2:** The parameters of the ultrasonic peening treatment.

Current/A	Frequency/KHz	Amplitude/µm	Time/min	Impact Needle Shape	Impact Position
3.5–4.0	19 ± 1	48	5	Circular	Weld toe

**Table 3:** Fatigue data of six samples.

Samples	Nominal Stress Range/MPa	Fatigue Life/Cycles	Peening Interval/Cycles
A	270	568,516	No supplement
B	270	1,074,959	100,000
C	270	627,278	150,000
D	255	878,512	No supplement
E	255	1,104,878	100,000
F	255	1,125,487	150,000
